# More attentional focusing through binaural beats: evidence from the global–local task

**DOI:** 10.1007/s00426-015-0727-0

**Published:** 2015-11-26

**Authors:** Lorenza S. Colzato, Hayley Barone, Roberta Sellaro, Bernhard Hommel

**Affiliations:** Cognitive Psychology Unit, Institute for Psychological Research and Leiden Institute for Brain and Cognition, Leiden University, Wassenaarseweg 52, 2333 AK Leiden, The Netherlands

## Abstract

A recent study showed that binaural beats have an impact on the efficiency of allocating attention over time. We were interested to see whether this impact affects attentional focusing or, even further, the top-down control over irrelevant information. Healthy adults listened to gamma-frequency (40 Hz) binaural beats, which are assumed to increase attentional concentration, or a constant tone of 340 Hz (control condition) for 3 min before and during a global–local task. While the size of the congruency effect (indicating the failure to suppress task-irrelevant information) was unaffected by the binaural beats, the global-precedence effect (reflecting attentional focusing) was considerably smaller after gamma-frequency binaural beats than after the control condition. Our findings suggest that high-frequency binaural beats bias the individual attentional processing style towards a reduced spotlight of attention.

## Introduction

When two beats of slightly different frequency (for instance 300 and 340 Hz) are presented separately to the left and right ears, the hearer detects a single beat that differs in amplitude at a frequency equal to the frequency difference between the two beats (40 Hz); a perceptual illusion known as the binaural auditory beat. While the neural mechanisms underlying this illusion are still unclear, very recent studies have shown that beat stimulation significantly affects functional brain connectivity (Gao et al., 2014) and modulates intracranial power and phase synchronization (Becher et al., 2015). These findings support the idea that the neural phase locking elicited by binaural beats can influence ongoing cognitive processing (Karino et al., 2006; see, Chaieb et al., 2015, for a recent review on the effect of binaural beats on cognition and mood). Low-frequency binaural beats are associated with mental relaxation and high frequency beats with alertness and attentional concentration (Vernon, 2009; Turow & Lane, 2011). This suggests that high-frequency beats might facilitate attentional control, which would fit with the observation that high-frequency neurofeedback training over the frontal cortex improves attentional efficiency (Keizer, Verment, & Hommel, 2010).

Very recently Reedijk, Bolders, Colzato, and Hommel (2015) have shown that binaural auditory beats affect how people control and monitor their visual attention. Participants listened to binaural beats while performing an attentional blink (AB) task, which assesses the efficiency of allocating attention over time. The size of the AB was considerably reduced by the binaural beats at least in some participants, which suggests that beats have a specific impact on how people allocate their attention over time. However, it is not yet clear how binaural beats affect attentional allocation over space. A well-known task that taps into this issue is the global–local task developed by Navon (1977). This task assesses how fast people can process global versus local characteristics of hierarchically constructed visual stimuli (e.g., larger shapes made of smaller shapes). Participants are typically confronted with a global stimulus (e.g., large rectangle) that is made of smaller shapes (the local stimuli), and the relationship between global and local stimuli can be congruent (e.g., a large rectangle composed of small rectangles) or incongruent (a large rectangle made of small squares). Typically, this task gives rise to the “global precedence” effect (i.e., performance is better when responding to global than to local features), which implies that global features can be processed faster than local features. Even though some models suppose that global and local level information is selected from associated spatial frequency channels (Robertson & Lamb, 1991) or by binding level information to feature-like representations of the hierarchical structure (Hübner & Volberg, 2005; Volberg & Hübner, 2004), global precedence is generally assumed to indicate a bias towards a large, comprehensive attentional focus, while attending to local features is considered to require more attentional effort.

There are reasons to assume that binaural beats might affect the size or scope of the attentional focus. Evidence for a role of individual differences in attentional control in the global–local task comes from Dale and Arnell (2010, 2015), who found a negative correlation between global precedence and AB magnitude: people who showed a smaller global precedence effect (i.e., a relatively stronger disposition towards processing local information) showed a greater AB magnitude. These observations suggest that individuals can exert control over the allocation of attention when processing targets. Interestingly, binaural beats might impact attention in a global–local task in two, not necessarily mutually exclusive ways.

First, processing global information is commonly assumed to require a broader, more spatially distributed focus of attention, while processing local information is assumed to rely on a smaller, more tightly controlled focus (Navon, 1977, 1981; Kimchi, 1992; Kimchi & Palmer, 1982). As high frequency beats arguably promote alertness and attentional concentration (Vernon, 2009; Turow & Lane, 2011), one might expect that listening to gamma-frequency (40 Hz) binaural beats is associated with a smaller global precedence effect than listening to a constant tone of 340 Hz (control condition). Indeed, if gamma-frequency binaural beats support the processing of local features, this should reduce the difference between performance on global and on local features. If this is the case, we would expect an interaction between the instructed target level (global vs. local) and the kind of beats (gamma-frequency vs. control). Theoretically, such an interaction would indicate a relatively direct impact of binaural beats on the focus or distribution of visual attention.

Second, the global–local task generates conflict by providing irrelevant information that indicates an alternative response. As pointed out previously, global–local tasks often employ congruent and incongruent stimuli, so as to prevent strategies that use information from one level to predict information from the other. Most importantly for our purpose, performance is better in congruent than in incongruent trials (Navon, 1977). This implies that adopting a global or local task set does not prevent the processing of information related to the other task, which can be taken to indicate a task or goal conflict (Kiesel et al., 2010). If we assume that high frequency beats promote alertness and attentional concentration (Vernon, 2009; Turow & Lane, 2011), we would predict that congruency effects are smaller when listening to gamma-frequency beats than while listening to a constant tone. If this is the case, we would expect an interaction between congruency and the kind of beats (gamma-frequency vs. control), with a smaller impact of congruency with gamma-frequency beats than with a constant tone. Theoretically, such an interaction would suggest a rather general impact of binaural beats on cognitive control, rather than a more specific impact on visual attention. Given that our previous study (Reedijk et al., 2015) has shown that the effect of binaural beats on AB magnitude was limited to the gamma-frequency binaural beats, we focused our investigation on the high-, but not low-frequency binaural beats.

## Method

### Participants

Thirty-six students (22 female, 14 male; aged 18–28 years old) from Leiden University participated in this study in exchange for course credit or pay. All had normal or corrected-to-normal sight and hearing. Participants were selected individually using the Mini International Neuropsychiatric Interview (MINI; Sheehan et al., 1998). The MINI is a well-established brief diagnostic tool in clinical, drug and stress research that screens for several psychiatric disorders and drug use (Sheehan et al., 1998; Colzato, Kool & Hommel, 2008, Colzato et al., 2011). Randomly, 18 participants (10 female, 8 male) were exposed to gamma-frequency (40 Hz) binaural beats session and 18 (12 female, 6 male) to a control condition (a constant tone of 340 Hz) session. Written informed consent was obtained from all subjects; the protocol and the remuneration arrangements of 5 euro were approved by the local ethical committee (Leiden University, Institute for Psychological Research).

### Global–local task

**Fig. 1:**
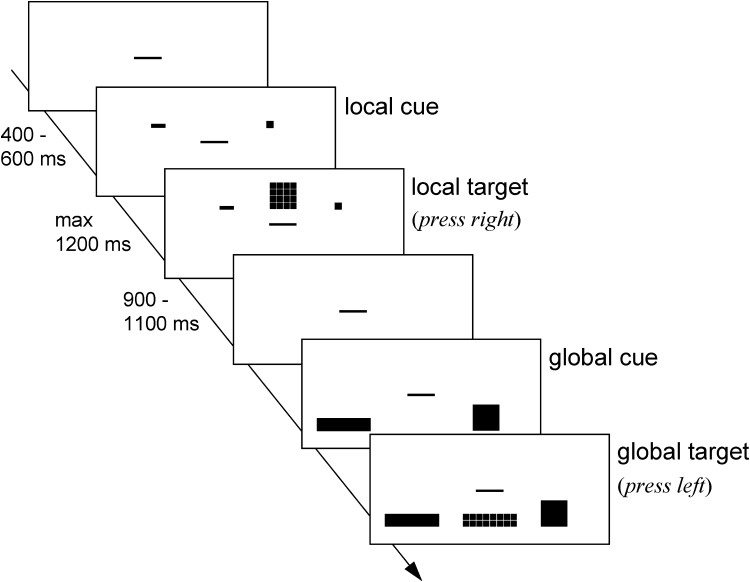
Sequence of events of a local trial followed by a global trial. Larger (global)* rectangles*/*squares* consisted of smaller (local) *rectangles* or *squares*. The congruent trial presents a bigger shape composed of similar smaller shapes (e.g., a *large square* consisting of *smaller squares*), the incongruent trial presents a bigger shape composed of different smaller shapes (e.g., a *large rectangle* consisting of *smaller squares*)

The task was adopted from Colzato, van den Wildenberg and Hommel (2008) and Steenbergen et al. (2015). The experiment was controlled by a Windows-operated computer attached to a Philips 17′′ monitor. Participants were seated at a viewing distance of 57 cm from the monitor (screen resolution: 1024 × 768; refresh rate: 100 Hz). Responses were made by pressing the “Z” or “/” of the QWERTY computer keyboard with the left and right index finger, respectively. The target stimuli consisted of hierarchical geometric figures, namely, larger (global) rectangles (1.5° × 6.4°) or squares (3.2° × 3.2°) composed of 16 smaller (local) rectangles (0.3° × 1.5°) or squares (0.7° × 0.7°). The space between the local elements of a stimulus was 0.1° both horizontally and vertically. In each trial, participants were presented with one of the four possible target stimuli: a rectangle consisting of smaller rectangles or squares, or a square consisting of smaller rectangles or squares. They had to alternate between responding to the local and to the global dimension of the target stimuli every four trials. The rectangle or square was associated with a spatially assigned response button that was pressed with either the left (“z” from computer keyboard) or right (“/” from computer keyboard) index finger; the stimulus–response mapping was randomized across participants. Target stimuli were presented either in the upper or in the lower part of the screen, depending on the to-be-attended level. Target stimuli presented in the upper part of the screen signaled to respond to the local (global) level, whereas those presented in the lower part of the screen signaled to respond to the global (local) level. The target position–local/global rule assignment varied randomly across participants. The to-be-attended level (global or local) was primed by a cue appearing 400–600 ms before the target stimulus (see Fig. 1). Cues that related to the global (local) dimension consisted of a big (small) square, presented at one side of the target stimulus, and a big (small) rectangle, presented at the other side of the target stimulus. The “local” and “global” cues were the same size as the global and local stimuli, and their left/right location corresponded to the position of the required response. Cues and target stimuli were presented in red on a white background, and both remained on the screen until a response was given or 2500 ms had passed. The interval between response and presentation of the next cue was 900–1100 ms (see Fig. 1).

The task consisted of a single experimental block of 160 trials, in which the four possible target stimuli were presented with equal probabilities. Therefore, participants performed on a total of 80 global trials and 80 local trials. Half of the trials were congruent (a large square consisting of smaller squares or a large rectangle consisting of smaller rectangles) and the other half of the trials were incongruent (a large square consisting of smaller rectangles or a large rectangle consisting of smaller squares). Moreover, of the resulting 160 trials, 39 included a task/level switch and 120 (not considering the very first trial) a task/level repetition. The experimental block was preceded by two training blocks of 50 trials each, in which the dimension to be attended (global or local) was constant across all trials within that block. Training block order was counterbalanced between participants, meaning that half of the participants started with the “local block,” the other half with the “global block.”

### Procedure

Participants were invited individually to the laboratory. In both sessions, upon arrival, they were asked to rate their mood on a 9 × 9 Pleasure × Arousal grid (Russell, Weis, & Mendelsohn, 1989) with values ranging from −4 to 4. Thus, the scale provides a score that indicates the location of the participant’s affective state within a two-dimensional space defined by hedonic tone and activation. Subsequently, participants listened to gamma-frequency (40 Hz) binaural beats or a constant tone of 340 Hz (control condition), all embedded in white noise to enhance clarity of the beats (Oster, 1973), for 3 min before and during the global–local task (training and experimental blocks). Binaural beats were presented through in-ear headphones (Etymotic Research ER-4B microPro), which provide 35 dB noise attenuation. Both binaural beat conditions were based on a 340 Hz carrier tone, which was used as the constant tone in the control condition. After the global–local task, participants rated their mood for the second time. After these measurements, the experimental session was ended and participants were paid, debriefed and dismissed.

### Statistical analysis

Mean RTs and proportions of errors were analyzed by means of ANOVAs using target level (global vs. local), the congruency between the stimuli on the two levels (congruent vs. incongruent), and task switch (i.e., same vs. different target level as in previous trial: task repetition vs. alternation) as within-subjects factor and group (gamma vs. control) as between-subject factor. Mood was analyzed by means of a repeated-measures analysis of variance (ANOVA) with effect of time (first vs. second measurement) as within-subjects factor and group (gamma vs. control) as between-subject factor. A significance level of *p* < .05 was adopted for all statistical tests.

## Results

### Global–local task

**Fig. 2:**
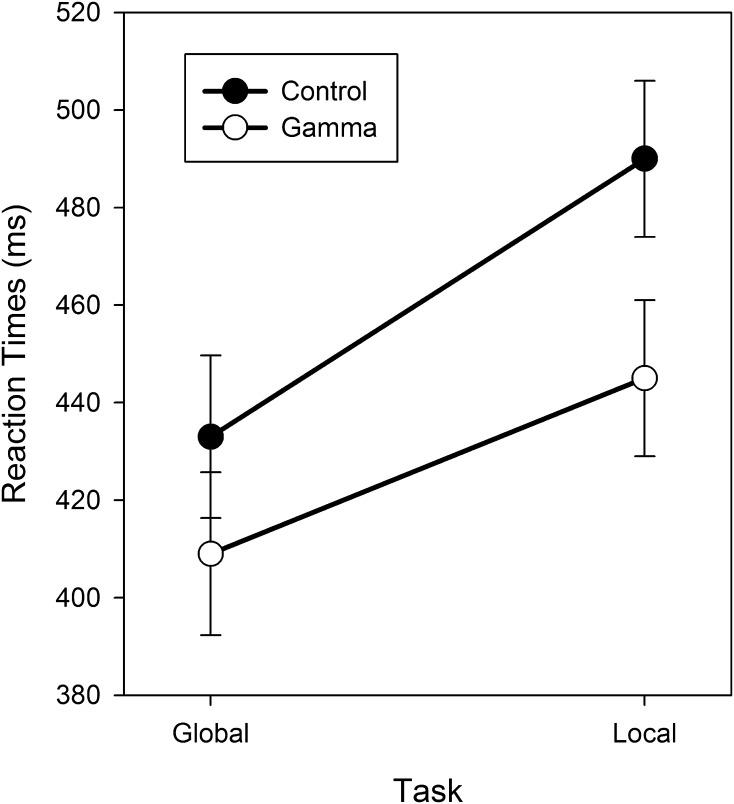
Mean reaction times (ms) as a function of group (gamma vs. control) and target level (global vs. local). *Error bars* represent standard errors

The reaction time analysis showed five significant sources of variance, see Table 1. First, the effect of target level, *F*(1,34) = 80.97, *p* < .0001, MSE = 1879.08, *η*_*p*_^2^ = 0.70, reflecting the well-known global precedence effect (Navon, 1977), that is, faster responses to globally than locally defined targets (421 vs. 467 ms). Second, the effect of congruency, *F*(1,34) = 31.70, *p* < .0001, MSE = 1502.73, *η*_*p*_^2^ = 0.48, reflecting interference of the irrelevant target level, as indicated by a faster RT on congruent as compared to incongruent trials (431 vs. 457 ms). Third, the effect of switching, *F*(1,34) = 27.59, *p* < .0001, MSE = 7429.21, *η*_*p*_^2^ = 0.45, which revealed that repeating the task allowed for faster responding than switching between target levels (418 vs. 471 ms). Fourth, the interaction of task switch and congruency, *F*(1,34) = 4.22, *p* < .05, MSE = 1459.05, *η*_*p*_^2^ = 0.11, indicated that the congruency effect was larger when target levels were switching (35 ms) than when they were repeated (17 ms). Most importantly for our purposes, target level interacted with group, *F*(1,34) = 4.21, *p* < .05, MSE = 1879.08, *η*_*p*_^2^ = 0.11 (see Fig. 2): the size of the global precedence effect was significantly smaller in the gamma group (36 ms) than in the control group (57 ms). Last, both switch and congruency did not interact significantly with group, *F*(1,34) = 1.61, *p* > .05, MSE = 7429.21, *η*_*p*_^2^ = 0.04 and *F* < 1, respectively. Given that conventional null-hypothesis significance testing (NHST) cannot be used to provide evidence in favor of the null hypothesis (*H*_0_), we calculated the Bayesian (posterior) probability associated with the occurrence of the null hypothesis [*p*(*H*_0_|*D*)] to validate the absence of any interaction between the factors group and congruency (crucial for our second hypothesis). To this end we used the method proposed by Wagenmakers (2007) and Masson (2011). This method uses Bayesian information criteria (BIC), calculated using a simple transformation of sum-of-squares values generated by the standard ANOVA, to estimate the Bayes factor and generate *p*(*H*_0_|*D*), assuming a “unit information prior” (for further details, see Kass & Wasserman, 1995; see also Jarosz & Wiley, 2014).

**Table 1:** Mean reaction times (RT; in ms) and errors (in %) for each condition in the global–local task-switching paradigm as a function of and group (control vs. gamma) are displayed

Group	Control		Gamma	
	Mean RT	Mean error	Mean RT	Mean error
Switch	495 (22.2)	8.2 (1.3)	447 (22.2)	5.6 (1.3)
Repetition	428 (11.2)	6.2 (0.9)	407 (11.2)	6.1 (0.9)
Switch cost	67 ms		40 ms	
Local target	490 (16.1)	8.8 (1.5)	445 (16.1)	6.3 (1.5)
Global target	433 (16.8)	5.6 (0.9)	409 (16.8)	5.5 (0.9)
Global precedence effect	57 ms		36 ms	
Incongruent	476 (18.3)	11.4 (1.4)	439 (18.3)	8.9 (1.4)
Congruent	448 (14.1)	3.0 (0.8)	415 (14.1)	2.8 (0.8)
Congruency effect	28 ms		24 ms	

Standard errors are shown in parentheses

The *p*(*H*_0_|*D*) provides the exact probability of the occurrence of *H*_0_. The analysis revealed that the *p*(*H*_0_|*D*) was 0.84, hence, on the basis of the guidelines proposed by Raftery (1995), represents positive evidence in favor of *H*_0_.

The analysis of the error rates revealed only a main effect of congruency, *F*(1,34) = 70.31, *p* < .0001, MSE = 53.59, *η*_*p*_^2^ = 0.67, reflecting interference from the irrelevant target level, as indicated by a smaller proportion of errors in congruent than incongruent trials (2.9 vs. 10.2 %).

### Mood and arousal

As expected, the ANOVA performed on participants’ mean mood and arousal rating revealed a significant interaction between time and group only for arousal, *F*(1,34) = 4.29, *p* < .05, MSE = 0.93, *η*_*p*_^2^ = 0.11, but not mood, *F* ≤ 1. LSD Fisher post hoc analyses revealed that, for the control group, arousal levels were comparable across the two measurements (arousal levels were 0.5 and 0.3, for the first and second measurement, respectively, *p* = .61). In contrast, for the gamma group, a significant difference between the two time points indicated an increase from the first to the second measurement (0.3 vs. 1.1, *p* = .02), suggesting that our manipulation worked as expected.

## Discussion

The findings of our study are straightforward. First, corroborated by Bayesian inference, there was no indication that the congruency effect would be affected by binaural beats. If we consider that congruency reflects crosstalk from a currently irrelevant task or stimulus dimension (Kiesel et al., 2010), our observation indicates that gamma-frequency binaural beats do not lead participants/people to further engage in the suppression of currently irrelevant information in working memory. Second, however, high-frequency binaural beats did have a significant impact on the global precedence effect: the precedence effect became smaller, suggesting that visual attention became more focused than in the control condition.

A possible explanation of how high-frequency binaural beats might enhance attentional focus could mean that listening to gamma binaural beats entrains gamma band activity in the brain. Increased activity in the gamma frequency band is typically associated with greater attentional investment (Keizer et al., 2010; Rieder, Rahm, Williams & Kaiser, 2011), which in turn is associated with a deeper attentional blink (Olivers & Nieuwenhuis, 2006) and reduced global precedence effect (Dale & Arnell 2010, 2015). One way to test this possible connection would be to entrain gamma activity through mechanisms/techniques other than binaural beats, which should have the same effect. If successful, this might provide an interesting avenue for supporting attentional control abilities in clinical populations suffering from attentional disorders, such as attention-deficit-/hyperactivity disorder.

As pointed out in the introduction, it has been also proposed that global and local level information is selected by binding level information to feature-like representations of the hierarchical structure (Hübner & Volberg, 2005; Volberg & Hübner, 2004). This idea is particularly intriguing given that previous studies have suggested that the underlying neural underpinnings of binding features in the visual domain is supported by transient increases in synchronization in the gamma frequency range (Colzato et al., 2004, 2005). Accordingly, it may be that high-frequency binaural beats enhance binding processes within the visual cortex by entraining synchronization frequencies in the gamma band (Engel & Singer, 2001).

It seems interesting to contrast the observations of the present study with those of Reedijk et al. (2015) on the one hand and of Dale and Arnell (2010, 2015) on the other. While Reedijk et al. (2015) found that binaural beats eliminated the AB at least in some individuals, we found that binaural beats are reducing the global precedence effect. This would seem to suggest a positive correlation between the global precedence effect and the AB, which does not fit with Dale and Arnell’s (2010, 2015) records of negative correlations. This apparent discrepancy might suggest that the mechanisms underlying the impact of binaural beats on the AB and those underlying the impact of binaural beats on the global precedence effect are not the same. For instance, it might be that the precedence effect is affected through gamma-entrainment while the AB is affected through providing a temporal reference frame. Given that the AB can be reduced by optimal preparation of neural control states (Gross et al., 2006), a reduction of temporal uncertainty should result in a smaller effect. If the presentation of binaural beats provides a regular background that helps to predict the temporal onset of targets in the AB task, reduced AB should indeed be observed. For the time being however, this must remain speculation.

Our investigation used a between-subjects design to avoid possible practice effects on task performance. One cannot rule out that the differences we found in the global–local task are actually due to pre-existing group differences in cognitive-control styles rather than due to binaural beats exposure. Therefore, follow-up studies should determine whether our findings can be replicated in a within-subject comparison using different version of the global–local task and other tasks suited for investigating the attentional focusing, such as a flanker paradigm with varying distances between target and flanker.

In any case, our findings bring convergent evidence to the idea that binaural beats can affect and enhance cognition (Lane et al., 1998; Reedijk et al., 2013, 2015). While this is encouraging for the purpose of behavioral optimization in healthy and clinical populations, future studies should investigate how long-lasting binaural beats-induced biases of attentional control are. In sum, this is the first study to demonstrate that binaural beats induce an individual processing style that modulates visual attention. As we have argued, beats in the gamma range achieve this effect by increasing attentional focusing rather than by suppressing currently irrelevant tasks and task-related information.
